# Ten-year antidepressant medication trajectories among people who exit paid work when aged 66–76 years: a population-based cohort study

**DOI:** 10.1007/s00127-026-03080-w

**Published:** 2026-04-10

**Authors:** Tea Lallukka, Kristina Alexanderson, Katalin Gémes, Kristin Farrants

**Affiliations:** 1 Department of Public Health, University of Helsinki, Helsinki, Finland; 2 Department of Clinical Neuroscience, Division of Insurance Medicine, Karolinska Institutet, Stockholm, SE-17177 Sweden

**Keywords:** Older employees, Nationwide, Sick-leave, Mental disorder, Socioeconomic

## Abstract

**Purpose:**

Exiting paid work can be associated with mental health changes. Antidepressant medication is an indicator for mental health, but its trajectories have mostly been studied in younger populations. However, the trajectories could be different among older adults, who are a rapidly growing population group. Therefore, our aim was to explore these trajectories among people who continued working until 65–70 years and whether sociodemographic factors and prior sickness absence (SA) and disability pension (DP) due to mental diagnosis (mental SADP) were associated with such trajectories.

**Methods:**

All residents of Sweden aged 65–70 years old in 2010, who were in paid work 2005–2011 and exited paid work 2012–2016 (*N* = 32,849, 39% women), were included. We used register microdata for five years before and after work exit and included sociodemographic factors at the exit year and mental SADP in the years prior to exit. We used group-based trajectory modelling to examine trajectories in antidepressant medication (ATC-code N06A) and multinomial logistic regression to study factors associated with each trajectory.

**Results:**

Altogether, 90% had no antidepressant medication. Among those with medication, 94% had constant and 6% increasing use. Mental SADP before work exit was the strongest determinant of belonging to both identified trajectories. Older age at exit was associated with a lower likelihood of belonging to the increasing use trajectory.

**Conclusion:**

Antidepressant medication patterns remained largely constant after work exit. Prior mental SADP was the most consistent determinant of antidepressant medication among people exiting work at an older age.

**Supplementary Information:**

The online version contains supplementary material available at https://doi.org/10.1007/s00127-026-03080-w.

## Introduction

Extending working life in people of higher ages is a topical concern due to the ongoing major demographic changes. It is of paramount importance that more people can continue longer in paid work and maintain good health, including mental health, also after their work exit. Indeed, the percentage of people continuing in paid work after the age of 65 years has increased [1], which has also been a goal of many governments.

Common mental disorder (CMD), and antidepressant medication use (as one of its common treatments) has increased drastically in European countries [2]. However, the development of antidepressant use could be different among older adults as compared to the general population. Indeed, some studies suggest that there might be undertreatment and decreased medication use among older adults [3–5]. In Sweden, antidepressant medication has been increasing, also among older adults, aged 65 or more, albeit less sharply than among young individuals [6]. With aging populations globally, it is important to gain new knowledge about how mental disorders develop in older age, before and after exit from paid work, and how sociodemographic and health-related factors associated with distinct developmental patterns.

During work life, people with mental disorders have a higher risk of sickness absence (SA), disability pension (DP), and exit earlier from paid work [7]. This might lead to health selection and might mean that there are distinct features of those who continue working. Furthermore, individuals who continued working might also differ from younger people regarding mental health in the years around their later exit from paid work, as well as regarding factors associated with such patterns. Therefore, results from previous studies among younger age groups cannot be directly generalized to older employees [8, 9]. As mental health disorders are a key challenge for extended working lives, we need more knowledge regarding which groups may maintain good mental health and in which groups mental health may decline in older people who continue in paid work.

Exit from paid work in older age (after the age of 65 usually for retirement) is a major life transition. After the exit, mental health might change, which could be reflected by an increasing or decreasing need for healthcare, e.g., regarding prescribed medication [2]. While for some, mental health may deteriorate, there are also notable potentially positive changes. For example, according to a Finnish study, retirement appears to have a positive effect on life satisfaction, particularly among those with sub-optimal health before their work exit [10]. Based on a systematic review and meta-analysis, the odds of depression is reduced by 20% after retirement [11]. The follow-up time of the included studies was 2–25 years, and there were more than a half a million of participants, aged 45–80 years in most of the study populations. However, previous studies have typically relied on self-reported information and focused on individuals retiring at younger ages, ages in which health selection to retirement might differ from that among individuals who could stay at paid work after normative retirement age.

A focus on workers who exit paid work after the normative pension age is thus needed and a study of their mental disorders before and after the exit is currently lacking at the population level. Previous research has even excluded people who continue in paid work after the normative retirement age [12]. Information about the trajectories of antidepressant medication before and after exit from paid work can provide a more in-depth understanding of extended work careers and social- and health-related determinants.

Because sociodemographic and socioeconomic factors are associated with CMD, these need to be considered [13]. Additionally, in Sweden almost half of all SA spells lasting longer than 14 days are due to mental diagnoses [14], thus, they may indicate CMDs before exit from paid work and contribute to the distinct development of mental health.

The aim of this study was, thus, to investigate trajectories of prescribed antidepressants purchases five years before and five years after exit from paid work, among people who were in paid work past the age of 65. Additionally, we studied if sociodemographic factors, and prior SADP due to mental diagnoses (mental SADP) were associated with belonging to any identified trajectories.

## Methods

A longitudinal 10-year population-based cohort study was conducted, using linked microdata from four nationwide administrative registers. We included all people who lived in Sweden and were aged 65–70 in 2010, who were in paid work at least during 2005–2011, exited paid work at some point during 2012–2016, and did not have income from work in 2016–2020. We excluded those who had not exited paid work by 2016 (i.e., by age 76 for the oldest included), and those who exited before 2011 (i.e., before age 66 for the youngest included) (Online Resource 1). Thus, all individuals were aged 66–76 when they exited paid work. These criteria were applied to establish a cohort of people who continue working past the normative retirement age in Sweden of 65. This cohort was followed up until 2020, emigration, or death, which ever came first. Being in paid work was defined as having an income from work of at least twice the price base amount, a yearly amount set by the Swedish government for indexing purposes. This amounted to a work income of at least 78 800–94 600 Swedish krona (SEK, approx. EUR 8370–9400), depending on the year. Year of work exit (T_0_) was defined as the first year with less work income than this. We have complete data for those who lived in Sweden, but if an individual died or emigrated during the follow-up, they were not included in the trajectory analyses after death/emigration. To make meaningful trajectories, at least three data points (here years of medication purchases) were requested. Thus, those who died or emigrated were in the cohort, provided they had information for at least three years before death or emigration. The final study population consisted of 32 849 individuals, 39% women. The mean age at exit from paid work was 70.06 years (95% CI 70.00–70.09) for these women and 70.50 years (95% CI 70.47–70.53) for these men.

### The Swedish system

There is no set retirement age in Sweden, nevertheless, the normative retirement age was 65 years in the study period. However, retirement age was flexible and could start as early as 61. Employees had the right to keep their permanent position until age 67, after which the choice to retain or terminate their employment was up to the employer. Despite the flexible pension system being designed to enable and encourage people to extend their working lives, a considerable number of people in Sweden retired before or at the age of 65 [15, 16]. Still, many continued with some type of paid work. One could draw pension at the same time as having paid work.

All people in Sweden with permanent work incapacity due to morbidity could claim DP up to age 65. All people with income from work, unemployment benefits, or parental benefits whose morbidity led to work incapacity could claim SA benefits. After age 65, individuals could claim SA for up to 180 days total. The Social Insurance Agency could grant more SA days, if they deemed it likely that the individual would return to work. After age 70, an additional 180 days could be granted, but no extension. Both SA and DP can be granted for full- or part-time of ordinary work hours. In Sweden, the patient’s costs for prescribed medication were reimbursed if the cost exceeded 2000 SEK (~ 180 EURO) during a 12-month period.

## Data sources

We used microdata from four nationwide administrative registers. Statistics Sweden’s Longitudinal integrated database for health insurance and labor market studies (LISA) [17] provided sociodemographic and socioeconomic variables (age, sex, income from work, country of birth, migration, type of living area, educational level, and family situation). The Swedish Social Insurance Agency’s MiDAS register provided information on SA (in spells > 14 days) and DP (regarding dates, length, and diagnoses). The Swedish National Board of Health and Welfare’s Cause of Death Register provided death dates. Finally, the Prescribed Drug Register provided dates and defined daily doses (DDD) of all prescribed dispended antidepressant medications (N06A).

The data were linked at individual level by Statistics Sweden, by use of the individual PIN code all people living in Sweden have [17, 18]. The data were analyzed pseudonymized. These administrative population-based datasets are of high quality and further details about the datasets are reported elsewhere [1, 17, 19–22].

## Variables

We used complete register data linkages comprising information on sociodemographic factors and prior SADP due to mental diagnoses to examine their associations with any identified trajectories of prescribed antidepressants purchases: sex (women, men), birth country (Sweden, not Sweden), educational level (elementary (< 10 years, including missing, *n* = 62), high school (10–12 years), university/college (> 12 years)), type of living area based on Eurostat’s Degree of Urbanization (DEGURBA) classification system (big cities, medium-sized cities, rural areas) [23, 24], family situation (married/cohabiting with or without children < 18 years at home and single with or without children), and age (66–70, > 70), all measured at the year of exit from paid work. SA and DP due to mental diagnosis (F00–F99 + Z73.0) during five years prior to exit from paid work was classified as yes or no. Because the proportion of individuals with SADP due to mental diagnoses (any F-codes, + Z73.0, ICD-10) was small, we used a binary indicator of having had any SADP to preserve power.

For our outcome, prescribed antidepressants purchases, we included the number of annual DDDs of antidepressants (N06A) medication purchases from the prescribed drug register. The DDDs were included from each year 2005 through 2020, and individual follow-up was centered around the exit year from paid work (T_0_). There were no missing data for any variables used in this study, except for educational level as mentioned above. Missing values for education usually refer to low education, which is why we combined the few missing with the elementary education group.

### Statistical analysis

Antidepressant medication purchases were used to model trajectories before and after work exit using group-based trajectory modelling (GBTM), and then average marginal effects (AME) to study factors that were associated with distinct developmental patterns. We used group-based trajectory analysis centered on year of exit from paid work (T_0_) and then created an individual follow-up of five years before and after the exit year (T_− 5_ to T_+ 5_). Individuals were censored at emigration or death. Model fit was chosen based on decreasing Bayesian Information Criterion (BIC), decreasing Akaike Information Criterion (AIC), that the average group probability should be > 0.9 in all groups, and that each trajectory group should contain at least 5% of the population used to model trajectories.

For those with no antidepressant medication purchases, their trajectory is not latent but could be directly observed from the data. Thus, GBTM was conducted among those with at least one purchase of antidepressant medication during the study period. We ran a series of group-based trajectory models to identify sub-groups within the population that followed distinct patterns in their antidepressant medication. A table showing the BIC, AIC, and average group probabilities for all models from 2 to 5 groups are available as Online Resource (Online Resource 2).

Multinomial logistic regression over group membership was adjusted for sex, age, country of birth, family situation, type of living area, educational level, all at T_0_, and having any sickness absence or disability pension days (SADP) due to mental diagnosis in the five years before T_0_. We reported AMEs as main results and odds ratios (OR) and their 95% confidence intervals as supplementary files for comparison (please see Online Resource 4). AMEs are recommended over odds ratios as they do not as easily exaggerate the associations, since group sizes do not affect the estimates in the same way as for odds ratios [25]. Additionally, a key advantage is that when using AMEs, we get estimates also for the reference trajectory; the largest group of those not using any antidepressants.

We used SAS 9.4 (www.sas.com) with the extension Proc traj for SAS version date February 24, 2020 (www.andrew.cmu.edu/user/bjones) for the trajectory analyses, R studio 4.3.1 (posit.co/products/open-source/rstudio) with packages Haven 2.5.3, Marginal effects 0.16.0, Tidyverse 2.0.0, Nnet 7.3–19 and Dplyr 2.3.4 to compute average marginal effects (AME), and SPSS version 28 (www.ibm.com/products/spss-statistics) for all other analyses. Data management was performed in SAS with some additional adjustments in SPSS for graph purposes.

## Sensitivity analyses

We also conducted some additional analyses to confirm and complement our findings. First, we displayed odds ratios and their confidence intervals in place of AMEs, to add to the interpretation about the associations between sociodemographic and health-related factors and trajectory membership. Secondly, SADP days were also analyzed using GBTM in place of the dichotomized variable, however, the number of individuals with SADP was too low to compute any meaningful trajectory groups among those with any SADP.

## Results

Table 1 shows the distribution of sociodemographic characteristics at the exit from paid work (T_0_). Approximately 40% of the study population were women, and 54% were 70 years or older at the time of exit. Additionally, over 90% were born in Sweden, and almost 40% had at least some university/college education. Most (> 60%) were married or cohabited, and about 40% lived in large cities, and 41% in medium-sized towns. Any mental SADP was found among 4% prior to their work exit. Antidepressant medication before the exit and during the exit year was found among 10% of the cohort, and the proportion was slightly lower after the exit (8%).

Table 2 shows the basic descriptive information (mean, median, maximum, minimum, standard deviation) on the development of DDDs among those who had any antidepressant medication during the study period. Both the mean and the median increased gradually each year until the final year, where there was a slight dip.

**Table 1:** Characteristics of the study population at the year of exit from paid work (T_0_)

		Numbers	%
Sex	Women	12,913	39.3%
	Men	19,936	60.7%
Age at exit from paid work	66–69 years	15,104	46.0%
	≥ 70 years	17,745	54.0%
Country of birth	Sweden	29,957	91.2%
	Not Sweden	2892	8.8%
Educational level	Elementary (< 10 years)	7396	22.5%
	High school (10–12 years)	12,465	37.9%
	University/ College (> 12 years)	12,988	39.5%
Family situation	Married/cohabitant	20,849	63.5%
	Single	12,000	36.5%
Type of living area	Big cities	13,014	39.6%
	Medium-sized cities	13,537	41.2%
	Rural areas	6298	19.2%
SADP due to mental diagnosis^a^	No mental SADP	31,445	95.7%
	Any mental SADP	1404	4.3%
Antidepressant medication before exit from paid work	No	29,498	89.8%
	Yes	3351	10.2%
Antidepressant medication in the year of exit from paid work	No	29,445	89.6%
	Yes	3404	10.4%
Antidepressant medication after exit from paid work	No	30,119	91.7%
	Yes	2730	8.3%

^a^ SADP, sickness absence or disability pension days due to mental diagnoses (F-codes + Z73.0, ICD-10) during five years before exit from paid work

**Table 2:** Annual descriptive information of antidepressant medication purchases (defined daily doses, DDD of N06A) among those with DDD > 0 at any time point (follow-up year)

Time^a^	Mean	Median	Maximum	Minimum	Standard Deviation
T_− 5_	301	228	2444	0	291
T_− 4_	312	260	3626	0	299
T_− 3_	316	255	3791	0	295
T_− 2_	326	294	2207	0	290
T_− 1_	339	294	2440	0	302
T_− 0_	343	297	2648	0	299
T_+ 1_	348	298	2174	0	290
T_+ 2_	355	300	2200	0	301
T_+ 3_	360	300	2274	0	309
T_+ 4_	353	300	2414	0	303
T_+ 5_	341	298	4222	0	305

^a^T_0_ is the year of exit from paid work; T_− 5_ is five years before the exit and T_+ 5_ is five years after the exit

**Fig. 1:**
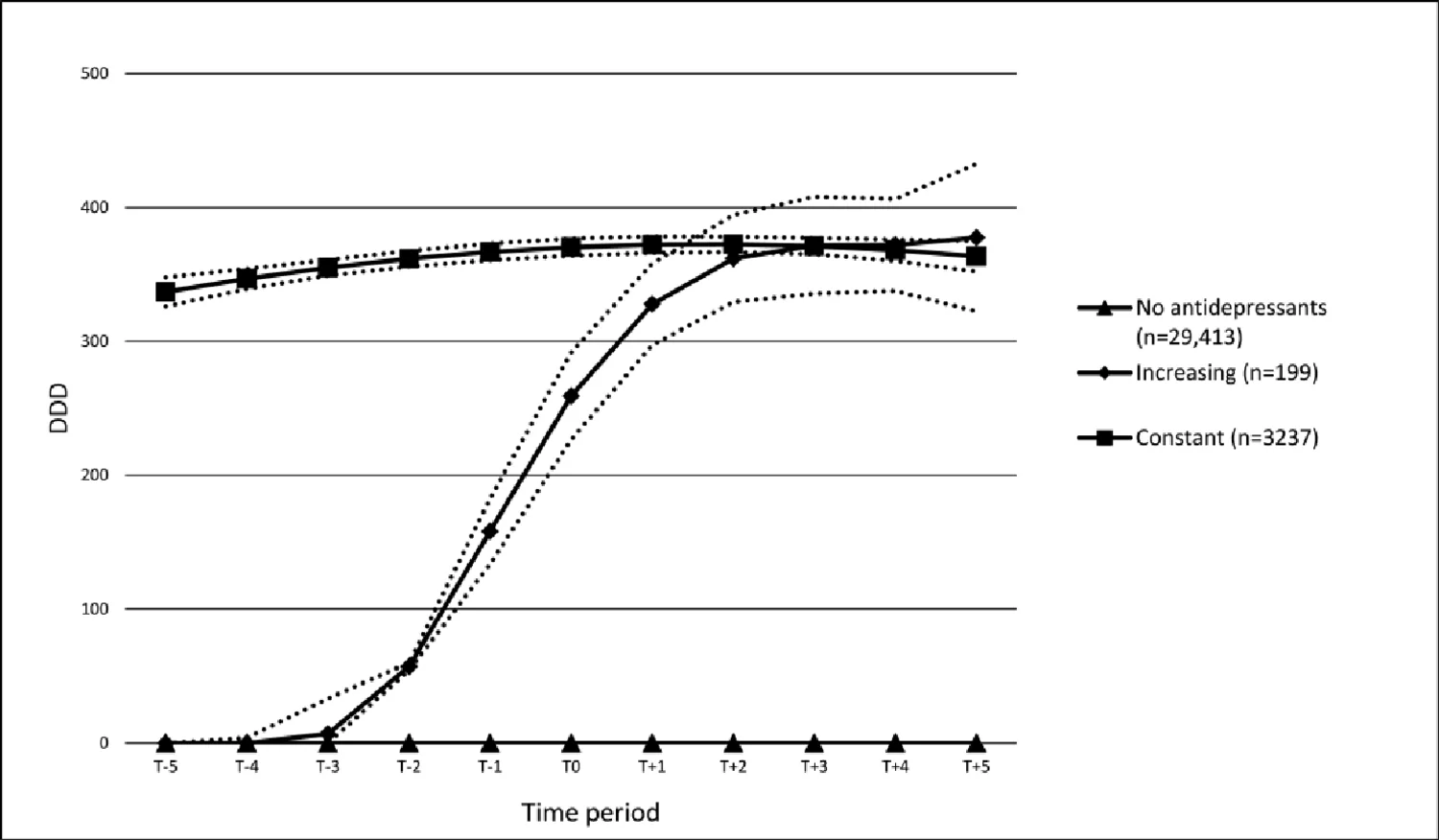
Antidepressant medication (defined daily doses, DDD, N06A) trajectories five years before and five years after exit from paid work (T_0_=exit), *n* = 32,849. Dashed lines represent the 95% confidence intervals

The largest group for the antidepressant medication outcome that was directly observed from the data, 89.6% of the total cohort, did not have any antidepressant medication during the 10-year study period. Thus, they formed the group ‘no antidepressant medication’ five years before or after their exit from paid work (reference). We selected a model with two latent groups describing antidepressant medication before and after exit from paid work (Fig. 1). Among those with antidepressant medication, 94.1% belonged to the group “Constant” (corresponding to 9.9% of the total cohort), and 5.8% to the group “Increasing” (corresponding to 0.6% of the total cohort), which started with 0 DDDs up until a few years before work exit (T_− 3_), when their use gradually increased to reach a stable trajectory at the same level as the constant medication use group in the years after exit from paid work at T_+ 2_ (Fig. 1). Online Resource 3 shows the estimated trajectory and the actual mean value of DDDs in trajectories with any antidepressant medication.

Table 3 shows the distribution of sociodemographic variables and prior mental SADP by each of the trajectory groups. There was a higher proportion of women in the “Constant” trajectory (53%) whereas the groups “None” and “Increasing”, respectively, had a higher proportion of men (62% and 64%, respectively). The group with the highest proportion of migrants was the “Increasing” group (17%, compared to 9% in “None” and 8% in “Constant”). There were only minor differences in the distribution of educational level and family situation between the trajectory groups. The proportion of individuals who had been on mental SADP was quite small in all groups, but still more than twice as high in the “Increasing” and “Constant” groups (10% and 12%, respectively) than in the “None” group (3%). There was a higher proportion of individuals who had been 66–69 years at exit in the “Increasing” group (61%) compared to in the “None” or “Constant” groups (46% in both groups).

**Table 3:** Study covariates (%) by trajectory of antidepressant medication purchases five years before and five years after the exit from paid work

		None	Increasing	Constant low
		*n* = 29,413	*n* = 199	*n* = 3237
Sex	Women	37.8%	36.2%	52.9%
	Men	62.2%	63.8%	47.1%
Age at exit from paid work	66–69 years	45.9%	61.3%	46.2%
	≥ 70 years	54.1%	38.7%	53.8%
Country of birth	Sweden	91.1%	82.9%	92.2%
	Not Sweden	8.9%	17.1%	7.8%
Educational level	University/ College (> 12 years)	39.3%	45.7%	41.1%
	High school (10–12 years)	38.0%	32.7%	38.0%
	Elementary (0–9 years)	22.7%	21.6%	20.9%
Family situation	Single	35.9%	38.2%	42.0%
	Married or cohabitant	64.1%	61.8%	58.0%
Type of living area	Big cities	39.5%	44.2%	40.7%
	Medium-sized cities	41.3%	42.2%	40.5%
	Rural areas	19.2%	13.6%	18.8%
SADP with mental diagnosis^a^	No mental SADP	96.6%	89.9%	88.2%
	Any mental SADP	3.4%	10.1%	11.8%

^a^ SADP, sickness absence or disability pension days due to mental diagnoses (F-codes + Z73.0, ICD-10) during five years before exit from paid work

Table 4 shows the AMEs for the associations between the examined sociodemographic and health-related factors and trajectory membership from the multinomial logistic regression expressed as percentage points with 95% confidence intervals (CI), both crude and fully-adjusted. Overall, the average marginal effects were quite small, with many differences being of a magnitude of a 1% point difference in likelihood or even less. This was especially true for the “Increasing” trajectory, which was very similar to the group with no antidepressant medication.

Sociodemographic factors generally did not have any strong associations with the trajectory membership. However, older age at work exit was associated with higher likelihood of belonging to the “Constant” trajectory, but a lower likelihood of belonging to the “Increasing” trajectory. Having had any mental SADP before exit from paid work was the most consistent determinant of belonging to the trajectories of “Constant” use of antidepressant medication, also after full adjustments, with 15% points higher likelihood of belonging to that trajectory than those without such SADP. Men were more likely to not have any antidepressant medication and women were more likely to belong to the “Constant” trajectory. Regarding odds ratios (OR), the strongest associations were similarly observed for having had any mental SADP when comparing individuals assigned to either the “Increasing“ group or the “Constant” group to individuals in the “None” group (Online Resource 4). Individuals having had any mental SADP compared to individuals with no mental SADP had higher OR to belong to the “Increasing“ group (OR 2.97, 95% CI 1.84–4.78) and to the Constant group (OR 3.33, 95% CI 2.93–3.78), as compared to the “None” group. In turn, individuals not born in Sweden had higher odds to belong to the “Increasing” group (OR 2.03, 95% CI 1.40–2.95) but lower odds to belong to the “Constant” group (OR 0.83, 95% CI 0.72–0.95), after full adjustments.

**Table 4:** Associations between sociodemographic and health-related covariates and trajectory group membership to one of the three trajectories. Average Marginal Effects expressed as % (95% confidence intervals) from the multinomial logistic regression models

		None		Increasing		Constant	
		Crude	Fully adjusted model^b^	Crude	Fully adjusted model^b^	Crude	Fully adjusted model^b^
Sex	Women	*ref*	*ref*	*ref*	*ref*	*ref*	*ref*
	Men	5.5 (4.8,6.2)	6.4 (5.4,7.5)	0.1 (-0.1,0.2)	0.4 (0,0.8)	-5.6 (-6.3,-4.9)	-6.8 (-7.9,-5.8)
Age at exit from paid work	66–69 years	*ref*	*ref*	*ref*	*ref*	*ref*	*ref*
	≥ 70 years	0.5 (-0.2,1.1)	-0.8 (-1.8,0.2)	-0.4 (-0.5,-0.2)	-0.7 (-1.1,-0.3)	-0.1 (-0.7,0.6)	1.5 (0.5,2.5)
Country of birth	Sweden	*ref*	*ref*	*ref*	*ref*	*ref*	*ref*
	Not Sweden	0.6 (-0.6,1.7)	1.7 (0.0,3.4)	0.6 (0.2,1.0)	0.9 (0.3,1.5)	-1.2 (-2.3,-0.1)	-2.6 (-4.2,-0.9)
Educational level	University/College (> 12 years)	*ref*	*ref*	*ref*	*ref*	*ref*	*ref*
	High school (10–12 years)	0.6 (-0.2,1.3)	0.0 (-1.1,1.2)	-0.2 (-0.4,0.0)	-0.3 (-0.7,0.1)	-0.4 (-1.1,0.4)	0.3 (-0.8,1.4)
	Elementary (< 10 years)	1.2 (0.3,2.1)	0.1 (-1.2,1.4)	-0.1 (-0.3,0.1)	-0.2 (-0.6,0.3)	-1.1 (-1.9,-0.2)	0.1 (-1.2,1.4)
Family situation	Single	*ref*	*ref*	*ref*	*ref*	*ref*	*ref*
	Married/cohabitant	2.4 (1.7,3.1)	1.0 (-0.1,2.0)	0.0 (-0.2,0.1)	0.0 (-0.4,0.3)	-2.3 (-3.0,-1.6)	-0.9 (-1.9,0.0)
Type of living area	Big cities	*ref*	*ref*	*ref*	*ref*	*ref*	*ref*
	Medium-sized cities	0.5 (-0.3,1.2)	0.2 (-0.9,1.3)	-0.1 (-0.2,0.1)	0.0 (-0.4,0.4)	-0.4 (-1.1,0.3)	-0.1 (-1.2,0.9)
	Rural areas	0.7 (-0.2,1.6)	0.5 (-0.9,1.9)	-0.2 (-0.5,0)	-0.4 (-0.9,0.1)	-0.4 (-1.3,0.5)	-0.1 (-1.4,1.3)
SADP with mental diagnosis^a^	No mental SADP	*ref*	*ref*	*ref*	*ref*	*ref*	*ref*
	Any mental SADP	-19.0 (-21.4,-16.6)	-16.5 (-18.8,-14.2)	0.9 (0.2,1.5)	1.1 (0.2,1.9)	18.1 (15.8,20.5)	15.4 (13.2,17.7)

^a^ SADP, sickness absence or disability pension days due to mental diagnoses (F-codes, + Z73.0, ICD-10) during five years before exit from paid work. ^b^ Fully adjusted for all variables simultaneously

## Discussion

### Main findings

In this large Swedish longitudinal cohort study, we aimed to identify distinct developmental patterns in antidepressant medication use five years before and after exit from paid work among those who exited after the age of 65 years. The main finding was that the exit from paid work was not associated with any major changes in the developmental patterns of antidepressant medication. Another main finding was that most individuals had no use of antidepressant medication before and after their exit from paid work. Among those with antidepressant medication, most had constant use, while for a very small percentage, the trajectory suggested increasing use. Prior mental SADP emerged as the most consistent determinant of the use of antidepressant medication even after the exit from paid work. Additionally, older age at exit was associated with a lower likelihood of belonging to the increasing use trajectory, but with a higher likelihood of belonging to the constant use trajectory.

### Interpretation and comparison to previous studies

Because previous studies on the area have focused on employees exiting paid work before age 65 [11], there are no directly comparable studies. Thus, comparisons must be made with caution, bearing in mind that previous results may not apply to those continuing to work past 65. However, as the rates of older people in paid work is growing [1, 26] this new information about their CMDs before and after their exit from paid work can be used to proactively estimate, e.g., medication cost and health and social care in terms of mental health needs. Many studies have also focused only on disability pension retirees, a population that differs from the current one in at least two notable ways: age at retirement and retiring due to morbidity, often a mental diagnosis [27, 28]. For example, in a previous population-based Swedish study, the age at inclusion was 19–64 and all had incident disability pension due to CMDs [27]. Thus, it is understandable that antidepressant medication was more common in that cohort as compared to in the current study, because a mental diagnosis was a prerequisite to be included. Another previous study, using Finnish data showed that those who experienced their jobs as stressful were more likely to have a decrease in their psychotropic medication use after retiring due to disability [28]. In addition to antidepressants (ATC code N06A), that study also included anxiolytics (NO5B) and hypnotics (NO5C). Because that study only included disability pension retirees, the mean age at retirement was much lower than in our study (52–56 years, depending on the diagnosis), limiting comparability.

Our main finding of the relatively stable developmental patterns of antidepressant use may indicate that the transition out of paid work is not associated with changes in CMDs among those exiting paid work late. One reason could be that access to healthcare does not change after exit from paid work. Moreover, all individuals in Sweden, irrespective of work status, have a ’high-cost limit’ to what they pay for healthcare and prescribed medication during a 12-month period. Importantly, it is of note that the majority of those exiting late did not have antidepressant medication. This is in line with a recent Finnish study, showing that a large proportion of old-age pensioners have good health both before and after work exit [29]. The result is also in line with a previous US study showing that chronological age does not sufficiently describe healthy aging and that a notable part of older people experience good health [30]. It is, thus, expected that a notable share of those who have continued in paid work past the age of 65 are experiencing good health and may continue to do so for an extended period after work exit. The antidepressant medication might also have helped alleviate different symptoms and therefore made it possible to continue in paid work and maintain well-being after the exit from paid work. However, we cannot confirm these speculations or interpretations with these data. With increasing age, health deterioration is common and expected biologically, however, many factors may affect this development. In our study, we focused on sociodemographic factors and prior SADP as possible factors contributing to the distinct developmental patterns. Indeed, results of a systematic review showed that positive effects on health after exit from paid work could merely or largely only concern those better off socioeconomically such as those with high education and better resources [13]. Also another systematic review highlighted the need to consider socioeconomic position, when assessing associations between exit from paid work and depression [31]. A study with data from several European countries suggested that with health policies aiming to extend work careers and working at older age, it is particularly relevant to consider that inequalities may widen when more individuals are able to work until older age [32]. This further highlights the importance of socioeconomic position, as benefits of working late and its consequences on subsequent development in health might differ by socioeconomic group. In all, the existing inequalities in health might be even widened. While we did not find sociodemographic factors to strongly contribute to distinct development in antidepressants, it does not mean that they are irrelevant. It is possible that those better off have been able to continue in paid work late. Alternatively, those with a poorer financial situation might have had to continue in paid work longer than they wished, however, we cannot confirm this in this cohort, selected based on age at exit from paid work. However, from other studies we know that there are large inequalities in health and differences in life expectancy in Sweden and throughout Europe [33–37].

In addition to sociodemographic factors, the associations between prior mental SADP and antidepressant trajectories were examined, to more comprehensively capture and identify mental health status before exit from paid work. While the strongest associations were found for this factor, the results were not surprising given the potential partial overlap between SA due to mental diagnoses and antidepressant use. Thus, many people on SA due to mental diagnosis would be assumed to have used or be using antidepressant medication, but they could also have other psychotropic medication or treatments, e.g., psychotherapy. On the one hand, people may have mental SADP for various reasons and *not* use medication. On the other hand, not all people with mental disorders have any SADP. In all, mental SADP prior to exit from paid work is likely to broadly reflect mental disorders [38]. This could explain its strong associations with the trajectories in antidepressant medication. As prior SA is a strong predictor of disability pension and early work exit [39, 40], many people with long SADP would not be included in our cohort of people working after age 65. Nevertheless, this variable remained the strongest predictor of future antidepressant medication. For those whose SADP was more directly work-related [41–43], one could have assumed that the use of antidepressant medication decreases after the exit from paid work. Contrary to this assumption, we did not observe any group that would indicate decreasing antidepressant medication. Such a group might exist but be too small to be detected separately. In turn, the small increasing trajectory might indicate a deteriorating mental health before exit from paid work. A plausible explanation could be that increasing common mental disorders lead to earlier exit from paid work, rather than the transition out of paid work leading to changes in antidepressant use among these older workers. However, we cannot confirm this assumption and as the group is small, strong conclusions are not warranted from this study, and the public health significance could be minor. Finally, the strong associations between mental SADP and antidepressant medication trajectory even after exit from paid work warrant more research and considering actions during work life to better support mental health of employees and prevent SA that may have long term consequences for health. In all, generalizing these results to other countries and contexts has to be done with caution. Sweden has high life expectancy and accessible good health and social care, and employees are entitled to long-term sickness absence benefits. They may thus have better opportunities to continue in paid work longer than in countries with very different systems. However, there is no particular reason to assume that the results would be notably different if health and social care and population age structures are relatively similar to those in Sweden. It is of note though that with increasing age and among the oldest old in particular, countries or cultural contexts, antidepressants may not be commonly used, or the need for them is missed, or other treatment options could be preferred [3–5, 44].

### Strengths and limitations

A key strength of this study is the use of comprehensive, large, nationwide, and high-quality register data, comprising of all people in Sweden fulfilling the inclusion criteria. There is no loss to follow-up. We could thus show the development of antidepressant medication use among the entire older population in paid work in Sweden, before and after exit from paid work. As the data are administrative, these data provide a more accurate picture of antidepressants use prevalence as compared to self-reported survey data with possible recall bias. This is of importance, since health status can notably affect both how symptoms and medication are reported as well as actual participation in surveys, where participants tend to be healthier as compared to target population [45]. A further advantage related to using antidepressants as the outcome is that by using such medications as an indication of a potential mental disorder, we also capture milder symptoms and a large population, not only the most severe disorders, requiring, e.g., secondary healthcare or hospitalization. Another aspect is that people who have experienced good effects of antidepressants for many years might be reluctant to reduce their medication. Furthermore, although we do not have a confirmed indication and antidepressant medication can be also used for insomnia or other reasons, antidepressant medication largely reflects depression and anxiety [46]. With register data it is also not possible to confirm if those who purchased the prescribed medication actually used them or if somebody else used them. This is unlikely a major issue as it is unlikely that people repeatedly pay for medication if they are not using them, and most had several purchases over the ten years. Based on these points and acknowledging some limitations, antidepressant medication can be assumed to be giving a relatively good indication of CMDs, especially in terms of depression and anxiety diagnosis, in the years before and after exit from paid work among older people in Sweden. It also needs to be noted that while antidepressants do not cover all medication for mental disorders, they are increasingly and commonly used [2], making them a relevant outcome both from the public health and societal point of views, e.g., due to their cost and reflecting the need for health and social care for prescriptions and other care.

One could also question how to define being in paid work; what income level to use for such definition, and what time frame without work income would best capture one’s work status. We estimated this based on income from paid work. Due to the limited data available to define work status, we cannot refer to retirement or confirm with certainty who is still in paid work. Future studies could examine other ways to define exit from paid work to confirm these results, and if the exit is largely for retirement as assumed. Our interest was on paid work exit, hence this is not a major limitation here.

As focus was on a cohort of older workers; those included might be somewhat more robust than others due to the known healthy worker effect [47, 48] or have had higher financial needs. However, with this approach we were able to show the trajectories of antidepressant medication among those who remained in paid work and that they still are distinct and may have partly different determinants. Using solely register-based data means that we, however, could not control for, e.g., key pertinent risk factors of mental disorders such as social relationships, loneliness, health behaviors, and obesity and consider their contributions to the distinct developmental patterns in antidepressant medication. Finally, a note regarding the use of group-based trajectory modelling is that they are but approximations of true development. Although the predicted probabilities and other statistics were very good for the latent trajectories, as our Online Resources display, some individuals can still have been misclassified into a group that does not describe their trajectory of the medication use. The number of trajectories identified is also affected by the selected software as our previous study showed, discussing limitations and strengths of using GBTM in more detail [49]. Finally, we acknowledge that comorbidity is possible, and it could be studied further, including its determinants.

## Conclusion

Most individuals (90%) who exited paid work after age 65 did not have any antidepressant medication, and the time of work exit did not mark a change in the developmental patterns in antidepressant medication, with one exception; a small group (0.6% of all; 6% of antidepressant medication users) increased their antidepressant medication. Thus, those who had such medication before work exit, continued the use after work exit, and no group was identified that would have started such medication after work exit or discontinued the use after work exit. Prior mental SADP was consistently associated with antidepressant medication trajectory, even after exit from paid work.

## Supplementary Information

Below is the link to the electronic supplementary material.


Supplementary Material 1


## Data Availability

The used data cannot be made publicly available due to privacy regulations. According to the General Data Protection Regulation, the Swedish law SFS 2018:218, the Swedish Data Protection Act, the Swedish Ethical Review Act, and the Public Access to Information and Secrecy Act, these types of sensitive data can only be made available for specific purposes that meets the criteria for access to this type of sensitive and confidential data as determined by a legal review. Contact imas-cns@ki.se for information regarding the data.
