# Supplementary material for: Ten-year antidepressant medication trajectories among people who exit paid work when aged 66–76 years: a population-based cohort study
Source: Soc Psychiatry Psychiatr Epidemiol. 2026 Apr 10;61(8):1591–602. doi: 10.1007/s00127-026-03080-w (PMC13521967; doi:10.1007/s00127-026-03080-w)
Supplement: Supplementary file 1 [file 127_2026_3080_MOESM1_ESM.pdf]

Online Resource Materials

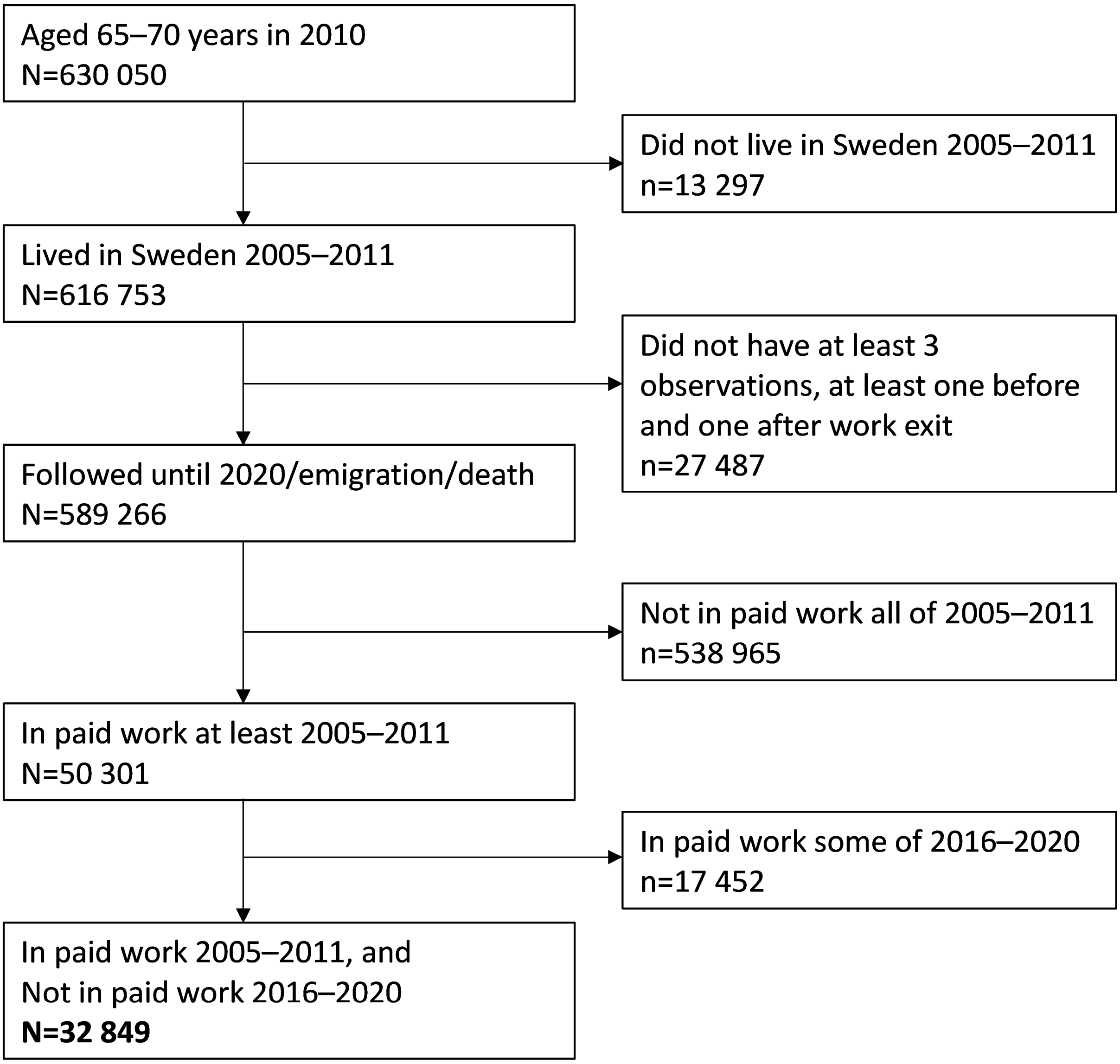

Online Resource 1. Flowchart of the study population

Online Resource 2. Model fit criteria (Bayesian Information Criterion, BIC and Akaike Information Criterion, AIC), and average probability of belonging to each group for 2–5 groups

DDD gt 0
(N=3436)
Adequacy tests

Type	Order				
2 groups cnorm	3 2	BIC= -8846.16 (N= 3436)		AIC= -8818.52	
3 groups cnorm	3 2 3	BIC= -8738.16 (N= 3436)		AIC= -8695.16	
4 groups cnorm	3 2 3 3	BIC= -8623.54 (N= 3436)		AIC= -8565.1	
5 groups cnorm	2 2 2 3 3	BIC= -8622.67 (N= 3436)		AIC= -8555.10	
Average group probabilities					
Model	Group 1	Group 2	Group 3	Group 4	Group 5
2 groups	0.95	1.00			
3 groups	0.95	0.99	0.82		
4 groups	0.95	0.97	0.84	0.81	
5 groups	0.93	0.85	0.99	0.88	0.81

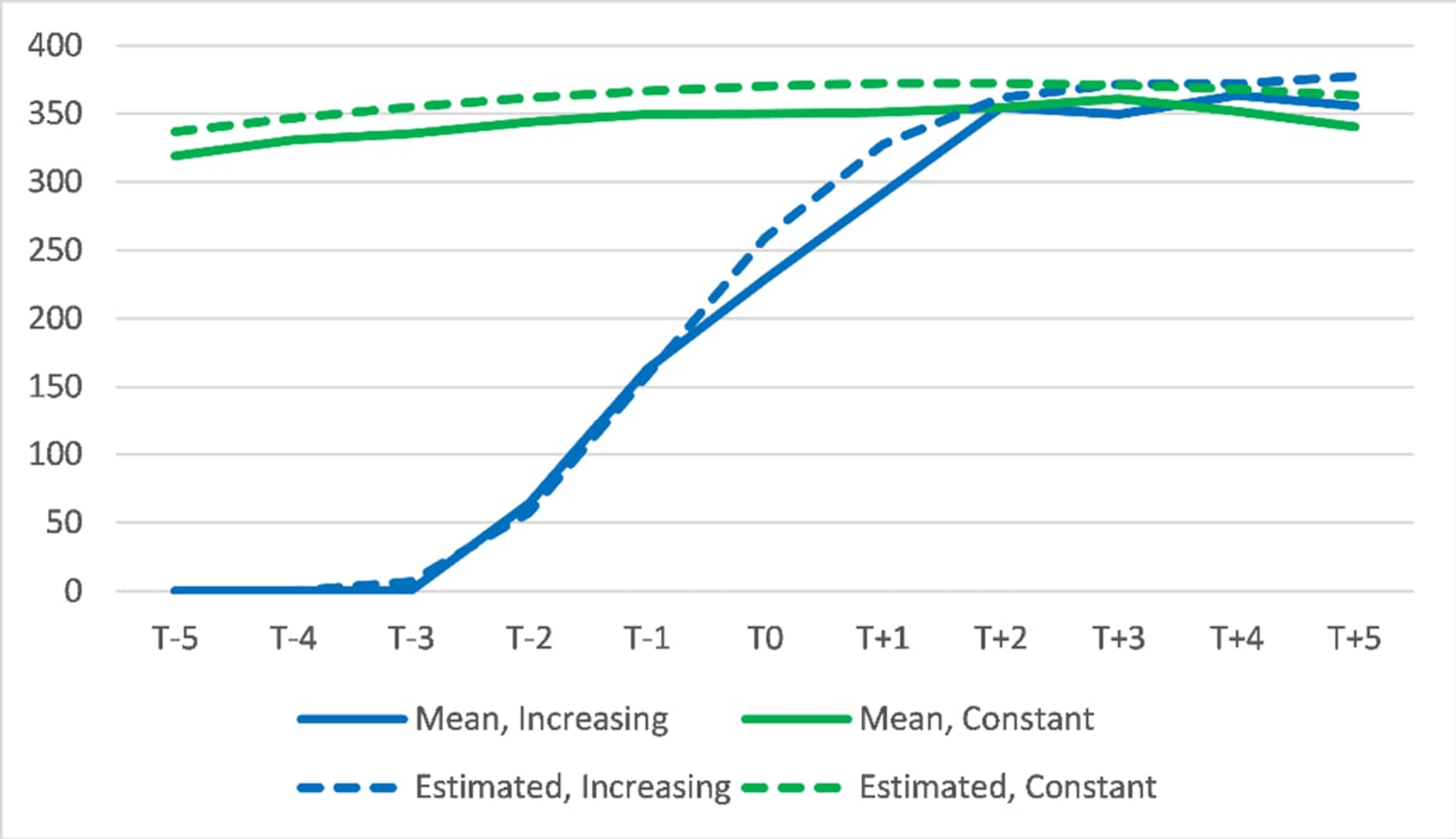

Online Resource 3. Actual mean values of antidepressant medication and the estimated trajectories (defined daily doses, DDDs, N06A) five years before and five years after the exit from paid work (T_0=exit)

Online Resource 4. Associations between sociodemographic and health-related covariates and trajectory of antidepressant medication before and after exit from paid work. Odds ratios (OR) from the multinomial logistic regression models (with 95% confidence intervals, low CI, high CI)

		None	Increasing			Constant		
Crude		*ref*	Est.	Low CI	High CI	Est.	Low CI	High CI
Sex	Women		1			1		
	Men		1.07	0.80	1.44	0.54	0.50	0.58
Age at exit from paid work	66–69 years		1			1		
	≥70 years		0.53	0.40	0.71	0.99	0.92	1.06
Country of birth	Sweden		1			1		
	Not Sweden		2.12	1.46	3.07	0.87	0.76	1.00
Educational level	University/ College (>12 years)		1			1		
	High school (10–12 years)		0.74	0.54	1.02	0.96	0.88	1.04
	Elementary (0–9 years)		0.82	0.57	1.18	0.88	0.80	0.97
Family situation	Single		1			1		
	Married or cohabitant		0.91	0.68	1.21	0.77	0.72	0.83
Type of living area	Big cities		1			1		
	Medium-sized cities		0.91	0.68	1.23	0.95	0.88	1.03
	Rural areas		0.63	0.41	0.97	0.95	0.86	1.05
SADP with mental diagnosis[a]	No mental SADP		1			1		
	Any mental SADP		3.17	1.99	5.05	3.79	3.35	4.30
Fully adjusted model								
Sex	Women		1			1		
	Men		1.31	0.96	1.77	0.59	0.54	0.63
Age at exit from paid work	66–69 years		1			1		
	≥70 years		0.54	0.41	0.72	1.11	1.03	1.20
Country of birth	Sweden		1			1		
	Not Sweden		2.03	1.40	2.95	0.83	0.72	0.95
Educational level	University/ College (>12 years)		1			1		

	High school (10–12 years)	0.76	0.55	1.05	1.02	0.94	1.11
	Elementary (0–9 years)	0.89	0.61	1.29	1.00	0.91	1.11
Family situation	Single	1			1		
	Married or cohabitant	0.97	0.72	1.30	0.93	0.86	1.00
Type of living area	Big cities	1			1		
	Medium-sized cities	0.98	0.72	1.32	0.99	0.91	1.07
	Rural areas	0.69	0.44	1.07	0.99	0.89	1.10
SADP with mental diagnosis[a]	No mental SADP	1			1		
	Any mental SADP	2.97	1.84	4.78	3.33	2.93	3.78

[a] SADP, sickness absence or disability pension days due to mental diagnoses (F-codes + Z73.0, ICD-10) during five years before exit from paid work
